# AXL Receptor Tyrosine Kinase as a Therapeutic Target in Hematological Malignancies: Focus on Multiple Myeloma

**DOI:** 10.3390/cancers11111727

**Published:** 2019-11-05

**Authors:** Siyang Yan, Niels Vandewalle, Nathan De Beule, Sylvia Faict, Ken Maes, Elke De Bruyne, Eline Menu, Karin Vanderkerken, Kim De Veirman

**Affiliations:** 1 Department of Hematology and Immunology, Myeloma Center Brussels, Vrije Universiteit Brussel, 1090 Brussel, Belgium; siyang.yan@vub.be (S.Y.); Niels.Vandewalle@vub.be (N.V.); nathan.debeule@uzbrussel.be (N.D.B.); Sylvia.faict@vub.be (S.F.); Ken.Maes@vub.be (K.M.); Elke.De.Bruyne@vub.be (E.D.B.); Eline.Menu@vub.be (E.M.); Karin.Vanderkerken@vub.be (K.V.); 2 Department of Hematology, Tianjin Medical University, Tianjin 300060, China

**Keywords:** AXL, hematological cancers, selective inhibitors, prognostic value

## Abstract

AXL belongs to the TAM (TYRO3, AXL, and MERTK) receptor family, a unique subfamily of the receptor tyrosine kinases. Their common ligand is growth arrest-specific protein 6 (GAS6). The GAS6/TAM signaling pathway regulates many important cell processes and plays an essential role in immunity, hemostasis, and erythropoiesis. In cancer, AXL overexpression and activation has been associated with cell proliferation, chemotherapy resistance, tumor angiogenesis, invasion, and metastasis; and has been correlated with a poor prognosis. In hematological malignancies, the expression and function of AXL is highly diverse, not only between the different tumor types but also in the surrounding tumor microenvironment. Most research and clinical evidence has been provided for AXL inhibitors in acute myeloid leukemia. However, recent studies also revealed an important role of AXL in lymphoid leukemia, lymphoma, and multiple myeloma. In this review, we summarize the basic functions of AXL in various cell types and the role of AXL in different hematological cancers, with a focus on AXL in the dormancy of multiple myeloma. In addition, we provide an update on the most promising AXL inhibitors currently in preclinical/clinical evaluation and discuss future perspectives in this emerging field.

## 1. Introduction

*AXL* (also known as *UFO*, *TYRO7*, *ARK*) was first detected as an unidentified transforming gene in two chronic myeloid leukemia patients in 1988 [1]. Tyrosine kinase receptor 3 (TYRO3), AXL receptor tyrosine kinase (AXL) and c-Mer proto-oncogene tyrosine kinase (MERTK) comprise a unique family of receptor tyrosine kinases and is referred to as the TAM (TYRO3/AXL/MERTK) family [2]. AXL is a driver of diverse cellular processes involved in cancer pathogenesis including proliferation, survival, migration, metastasis, dormancy and chemoresistance. Increased *AXL* expression has been detected in a variety of solid tumors (e.g., prostate cancer, breast cancer, osteosarcoma, etc.) and hematological malignancies including chronic lymphocytic leukemia, chronic myeloid leukemia, acute myeloid leukemia, multiple myeloma and mantle cell lymphoma.

In this review, we will discuss the basic function of AXL, its major role in controlling the immune system in a normal and cancerous setting, how it contributes to cancer pathogenesis and specifically focus on the role of AXL in hematological malignancies. A summary of available AXL inhibitors, of which the majority are non-specific multi-kinase inhibitors, in a preclinical or clinical setting for hematological cancers will be provided as well.

## 2. Normal Biology of the AXL Receptor

### 2.1. The Basic Function of AXL and GAS6

The TAM receptors have their characteristic structures, with two immunoglobulin-like domains and two fibronectin-type III domains in the extracellular region and a distinctive intracellular kinase domain [2,3,4]. Growth arrest-specific protein 6 (GAS6) and Protein S (PROS1) are both members of the vitamin K-dependent protein family, sharing 42% amino acid identity and acting both as agonists for the TAM family [4,5,6]. GAS6 can deliver signal through all three TAM receptors (AXL > TYRO3 >>> MERTK), while PROS1 only has a binding affinity to MERTK and TYRO3 [6,7]. GAS6 has been detected in various tissues and cell types including heart, lung, kidney, intestine, bone marrow, endothelial cells, vascular smooth muscle cells, monocytes, and liver [7,8,9].

AXL/GAS6 signaling requires the binding of GAS6 to the extracellular domain, receptor dimerization, and subsequent autophosphorylation of the tyrosine residues within the cytoplasmic domain including Y-779, Y-821, and Y-866 [10]. GAS6-dependent AXL activation plays a role in various processes such as endothelial cell survival, natural killer cell development, hepatic regeneration, neuron migration and survival, platelet activation, and hematopoiesis [11,12,13,14,15].

### 2.2. The Role of AXL in the Immune System

**Figure 1:**
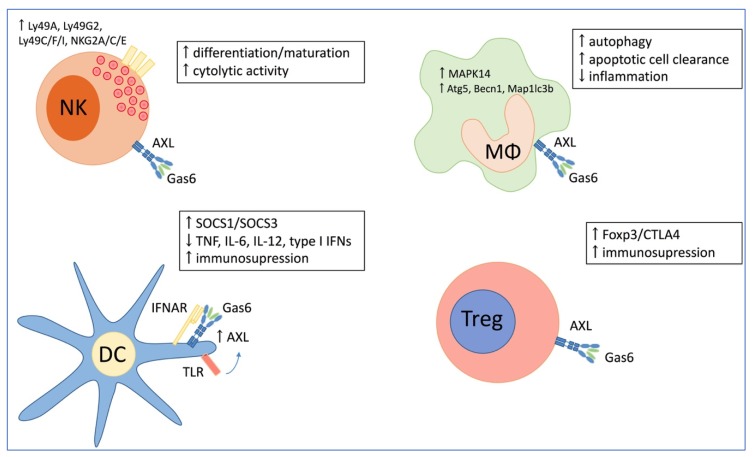
Schematic overview of GAS6/AXL expression in normal immunological cells including natural killer (NK) cells, dendritic cells (DC), regulatory T cells (Treg), and macrophages (MΦ). GAS6/AXL interaction regulates normal NK cell development, maturation and cytolytic activity. Binding of GAS6 to AXL increases the immunosuppressive capacity of DC and Treg. In macrophages, GAS6/AXL signaling induces autophagy, decreases inflammation, and regulates apoptotic cell clearance. Gas6 = growth arrest-specific protein 6.

TAM receptors and their ligands are predominantly expressed in macrophages, dendritic cells, monocytes and natural killer (NK) cells (Figure 1) [16]. Depending on the organ and cell type, AXL signaling regulates tissue repair, and pro- and anti-inflammatory immune responses [16]. The AXL/GAS6 pathway regulates normal NK-cell development and functional NK-cell maturation [13]. As part of the innate immune response, NK cells need to recognize and kill infected cells. The recognition and target killing rely on the expression of a group of inhibitory and activating NK cell receptors, whose expression requires TAM signaling [13,17]. Recombinant GAS6 and agonistic anti-AXL antibody upregulated the expression of NK cell-specific receptors (e.g., *Ly49A, Ly49G2, Ly49C/F/I, NKG2A/C/E*) and NK-cell associated genes (e.g., *IL-2Rβ*, *perforin*, *IL-15Rα*) in murine derived primary NK cell precursors (CD122+ NK1.1−DX5−) [18]. NK cells from TAM-deficient mice have very poor cytotoxic activity, exhibiting a 10-fold lower killing ability against target cells than normal NK cells [17]. AXL−/− mice have a lower number of NK cells and less NK cells could be generated in vitro from bone marrow derived hematopoietic stem cells indicating the importance of AXL in NK cell development and differentiation. In addition, it has been found that IL-15 plays an essential role in the differentiation of NK precursor cells into immature NK cells [19].

Dendritic cells have a modest AXL expression before pathogen encounter. Toll like receptor (TLR) activation and type I interferons (IFNs) strongly induce AXL expression through Janus kinase/signal transducers and activators of transcription 1 (JAK/STAT1) signaling [16]. The increased AXL proteins bind with the type I IFN receptors (IFNAR) and switch the pro-inflammatory state into an immunosuppressive response by activation of the suppressor of cytokine signaling (SOCS) 1 and 3 in dendritic cells. Through this mechanism, TAM receptors can inhibit TLR-induced production of pro-inflammatory cytokines, including tumor necrosis factor (TNF), interleukin-6 (IL-6), IL-12, and type I IFNs. Scutura et al. demonstrated that GAS6 stimulates migration and inhibits apoptosis of human dendritic cells via AXL activation [20].

In macrophages, AXL signaling is described to inhibit inflammation through autophagy [21]. Ligand-receptor interaction activates the p38α mitogen-activated protein kinase (MAPK14) pathway, resulting in increased expression of autophagy related 5 (*Atg5*), Beclin 1 (*Becn1*) and microtubule-associated proteins 1A/1B light chain 3B (*Map1lc3b*). GAS6/AXL-induced autophagy restrains the NLRP3 (NOD-, LRR- and pyrin domain-containing protein 3) inflammasome and in that way inhibits inflammatory responses. In addition, AXL plays a central role in apoptotic cell clearance by macrophages during inflammation. Inflammatory stimuli, including IFNα and the TLR3 ligand poly(I:C), induce AXL expression in murine and human macrophages, promote binding of AXL to GAS6, and increase macrophage capacity to engulf apoptotic cells [22,23].

Naturally occurring regulatory T cells (CD4+CD25+) are essential for maintaining immunological self-tolerance and immune homeostasis. Zhao et al. demonstrated that GAS6 increases the suppressive function of regulatory T cells in vitro and in mice, mainly through AXL activation [24]. GAS6–AXL binding induces the expression of forkhead box P3 (Foxp3) and cytotoxic T-lymphocyte associated protein 4 (CTLA4), proteins that contribute to the suppressor function of regulatory T cells.

## 3. The Role of AXL in Solid Cancers

### 3.1. Direct Effect of AXL on Tumor Growth

**Figure 2:**
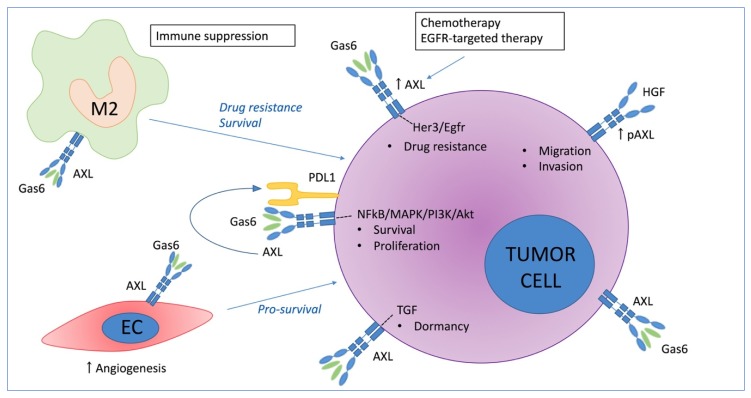
Schematic overview of the role of the GAS6/AXL axis in the tumor microenvironment focusing on tumor cell survival, proliferation, M2 polarization, migration, invasion, angiogenesis, dormancy, and drug resistance. EC = endothelial cells, M2 = type 2 macrophages. Her = human epidermal growth factor receptor, Egfr = epidermal growth factor receptor, HGF = hepatocyte growth factor, NFkB = nuclear factor kappa-light-chain-enhancer of activated B cells, MAPK= mitogen-activated protein kinases, Akt = protein kinase B, TGF = transforming growth factor, PDL1 = programmed death-ligand 1.

AXL phosphorylation and activation by binding of GAS6 has been associated with increased cell proliferation and survival in various tissues and cancer types including prostate cancer [25,26], colorectal cancer [27], gastric cancer [28], renal carcinoma and osteosarcoma [29,30]. AXL activation has been linked with distinct survival-associated signaling pathways including the nuclear factor kappa-light-chain-enhancer of activated B cells (NF-kB) pathway, mitogen-activated protein (MAP) kinases and phosphatidylinositol 3-OH kinase (PI3K) signaling and its downstream targets protein kinase B (PKB, also known as Akt) and S6 kinase (S6K) (Figure 2) [31,32,33]. In osteosarcoma cells, GAS6-induced AXL activation protected serum starved cancer cells from apoptosis, whereas knockdown of AXL inhibited cell proliferation and increased apoptosis [34]. In prostate cancer, microarray analysis and functional assays revealed that AXL is a critical mediator of cell survival via activation of the Akt-NF-κB signaling pathway [25].

Aberrant AXL expression also affects migration and invasiveness of cancer cells. Transfection of a dominant negative Axl (Axl-DN), which lack the kinase domain, significantly reduced motility, filopodia and cell-to-cell interactions in glioblastoma cells [35]. On the other hand, elevated AXL expression correlated with increased adherence, motility, and invasiveness of osteosarcoma cell lines [36]. More recently, Revach et al. demonstrated that high AXL expression in melanoma cells significantly enhanced invadopodia formation and function, which in turn is associated with the invasive phenotype of metastatic cancer cells [37]. In addition, it has been demonstrated that hepatocyte growth factor (HGF) induces AXL phosphorylation, independent of GAS6, and that the Met-AXL-Elmo2-Dock180 complex is critical for HGF-induced migration of cancer cells [38]. Duan et al. developed a GAS6/AXL blocking antibody, named DAXL-88, and observed reduced cancer cell migration and invasion in vitro [39].

### 3.2. The Role of AXL in Chemoresistance

Besides its role in survival, proliferation, and migration; AXL expression is a common resistance mechanism to immunotherapeutic, chemotherapeutic and molecular targeted agents. Hong et al. [40] found that AXL expression could be induced by chemotherapeutic agents including doxorubicin, etoposide (VP-16), and cisplatin; and resulted in activation of the AKT and extracellular signal–regulated kinases 1/2 (ERK1/2) survival pathways. In breast cancer, AXL was associated with doxorubicin-resistance through AKT/GSK-3β/β-catenin activation [41]. In ovarian cancer, AXL expression was significantly higher in chemoresistant ovarian tumors, compared to chemosensitive cancers [42]. Lentiviral knockdown of AXL expression in metastatic ovarian cancer cell lines increased sensitivity to the chemotherapeutic agents paclitaxel and carboplatin in vitro [42]. AXL inhibition by BGB324 (R428/Bemcentinib) increased response to chemotherapy in a patient-derived xenograft model from a chemoresistant ovarian cancer. D’Errico et al. demonstrated that TAM-mediated chemotherapy resistance is mediated by the 14-3-3ζ/AXL signaling pathway in pancreatic ductal adenocarcinoma [43].

AXL overexpression also mediates resistance to various targeted therapies including fms like tyrosine kinase 3 (FLT3, also known as CD135)-targeted therapies [44], phosphoinositide 3-kinases (PI3Ka) inhibitors [45,46], and epidermal growth factor receptor (EGFR)-targeted therapies (e.g., erlotinib, osimertinib, cetuximab) [47,48,49]. It has been demonstrated that the kinase activity of AXL is required for erlotinib resistance in EGFR-mutant non-small cell lung cancer (NSCLC) models [49]. Recently, Taniguchi et al. demonstrated that osimertinib-treatment increased AXL expression, causing cancer cell survival by receptor tyrosine-protein kinase erbB-3, also known as HER3, and EGFR re-activation and osimertinib tolerance. AXL inhibition by NPS1034 resensitized the cells to osimertinib treatment and resulted in reduced tumor size and delayed tumor growth compared to osimertinib alone [48]. Furthermore, AXL expression was sufficient to mediate acquired resistance to cetuximab in models of non-small cell lung cancer (NSCLC) and head and neck squamous cell carcinoma (HNSCC) [47]. Elkabets et al. [46] found that AXL mediates resistance to PI3Kα inhibition by activating the EGFR/PKC/mTOR axis in head and neck and esophageal squamous cell carcinomas.

In contrast to its role in proliferation, AXL may control quiescence and cancer cell dormancy. In prostate cancer, it has been demonstrated that GAS6/AXL and transforming growth factor-beta 2 (TGF-β2) signaling regulates tumor cell dormancy. Osteoblasts significantly induced AXL expression and decreased proliferation as determined by bromodeoxyuridine (BrdU) and Ki67 staining [50]. On the other hand, AXL knockdown induced proliferation of prostate cancer cells in the presence of osteoblasts. More recently, Axelrod et al. confirmed that AXL could indeed induce dormancy of prostate cancer cells; however it was insufficient to maintain long-term cellular dormancy [51].

### 3.3. Immune Mediated Pro-Tumoral Effect of AXL

As mentioned above, AXL is expressed in different immune cell populations including macrophages, natural killer cells, and regulatory T cells (Figure 1). AXL has been identified as a mediator of immunosuppression, however its specific role in the different immune subsets is still unclear. AXL and GAS6 are both described to be involved in the polarization of macrophages into an immunosuppressive M2 phenotype [52]. Incubation of THP-1 macrophages with tumor cell derived conditioned medium significantly increased M2 polarization and AXL expression, while AXL inhibition could abrogate M2 polarization. AXL inhibition by SLC-391 decreased tumor growth and increased the ratio of M1/M2-polarized macrophages in a CT26 murine colon carcinoma model [53].

AXL is also described to drive immune checkpoint signaling including programmed death-ligand 1 (PD-L1) [54]. AXL expression correlated with PD-L1 expression in lung adenocarcinomas and AXL targeting significantly reduced PD-L1 gene expression in the tumor cells [55]. Synergistic effects were observed in ID8 tumor-bearing mice, a model of ovarian cancer, when anti-PD1 antibodies and AXL inhibitors were combined (R428 and SGI-7079) [56].

These observations were recently confirmed by Yokoyama et al. who demonstrated that RXDX-106, a novel potent pan-TAM RTK inhibitor, reduced tumor growth by affecting the immune response instead of direct anti-cancer effects as demonstrated by the use of immune deficient and immunocompetent mice [57]. RXDX-106 treatment resulted in increased M1-polarized macrophages and an activation of natural killer cells. In addition, RXDX-106 in combination with anti-PD1 had more significant anti-tumor effects when combined compared to single agent treatment.

## 4. The Role of AXL in Multiple Myeloma

### 4.1. Current Treatment Strategies in Multiple Myeloma

Multiple Myeloma (MM) is an incurable malignancy of terminally differentiated plasma cells, residing primarily in the bone marrow, and is classified as the second most common hematological cancer. The cause for acquiring MM is idiopathic, although the disease is in all cases preceded by precursor states termed monoclonal gammopathy of undetermined significance (MGUS) or smoldering myeloma (SMM) [58]. The interaction of MGUS cells with cells of the bone marrow niche including immune cells, bone cells, and others may be key regulators of malignant transformation.

Treatment strategies for MM have changed dramatically over the past years, not only by the discovery of new classes of therapeutic agents, but also by defining more individual treatment schemes based on cytogenetics of the disease and personal characteristics of each patient [59]. At least six different classes of approved agents are available including proteasome inhibitors (e.g., bortezomib, carfilzomib), immunemodulatory drugs (IMiDs) (e.g., lenalidomide, pomalidomide), histone deacetylase inhibitors (e.g., panobinostat), monoclonal antibodies (e.g., daratumumab, elotuzumab), alkylating agents (e.g., melphalan), and autologous stem cell transplation; combined as doublet, triplet, or even quadruplet therapies [60]. Only a small fraction of patients manage to achieve a longstanding disease response with current treatment regimens. Ultimately, most patients relapse and succumb to this life-threatening and debilitating disease, indicating the need for novel compounds and therapeutic approaches.

### 4.2. TAM Receptors in Multiple Myeloma

Waizenegger et al. investigated the expression of all TAM receptors and its ligand GAS6 in patient-derived MM samples and myeloma cell lines [61]. The number of GAS6+ and MERTK+ bone marrow plasma cells (BMPCs) were significantly increased in myeloma patients compared to healthy controls; whereas AXL and TYRO3 were mainly undetectable in BMPCs of both groups and also in human myeloma cell lines. GAS6 promoted survival and proliferation of myeloma cells and protected them against bortezomib-induced apoptosis. In vivo, GAS6 overexpression shortened the overall survival of U266 orthotopic MM mice, while targeting of GAS6 by warfarin significantly increased survival and reduced tumor growth in this model. Mertk-knockdown by shRNA reduced survival and proliferation of myeloma cells, while Axl- or Tyro3-knockdown did not affect myeloma cell proliferation. All these data indicate that MERTK is the most important TAM receptor in proliferating myeloma cells and that GAS6/MERTK signaling regulates myeloma cell survival and resistance. Recently, small molecule MERTK-inhibitor R992 demonstrated anti-myeloma activity in vitro and in vivo [62]. MERTK blockade decreased proliferation and induced apoptosis of human myeloma cell lines. In addition, combination treatment with bortezomib and cyclophosphamide demonstrated that R992 inhibition significantly increased the chemosensitivity to these MM therapies. In vivo, R992 significantly reduced tumor load in the U266 systemic myeloma model. Moreover, R992 directly inhibited osteoclast differentiation and increased osteoblast differentiation, indicating that MERTK blockade not only affects the tumor cells but also reduces the associated bone disease.

### 4.3. AXL Expression in Multiple Myeloma

Recently, Khoo et al. described the expression of AXL in dormant myeloma cells. They were the first to develop an in vivo dormancy MM model using 5TGM1-eGFP murine myeloma cells labeled with a fluorescent membrane dye (Vybrant DiD) [63]. By intravital imaging, the presence of dormant MM cells could be investigated and both dormant and proliferating MM cells could be FACS sorted and characterized by single cell RNA sequencing. Dormant MM cells possessed a distinct transcriptome signature including increased Axl expression compared to proliferating MM cells. They demonstrated that Axl, macrophage-expressed gene 1 (*Mpeg1*), and signal regulatory protein α (*Sirpα*) expression could be induced by direct contact with osteoblasts in vitro. In vivo targeting of AXL with BMS-777607 or cabozantinib significantly reduced the number of dormant MM cells and increased proliferating MM cells, indicating that AXL inhibition releases MM cells from dormancy and sensitizes them to chemotherapy.

In contrast to Waizenegger et al., they observed that Axl was expressed in normal CD138+ BMPCs and is increased in plasma cells from patients with MGUS compared to plasma cells from overt MM and relapsed MM, suggesting that Axl decreases with disease progression [63]. Axl was not expressed in memory B-cells, in vitro generated polyclonal plasmablasts or human MM cell lines, but was detectable in osteoblasts and mesenchymal stem cells; indicating its importance in the surrounding bone marrow microenvironment. By nearest-neighbor analysis, they identified Axl-corelated genes (including *Vcam1*, *Vsig4*, *CD136*, *Apoe*, etc.) which could be used to discriminate control subjects and patients with MGUS, from patients with MM. Data suggested that the dormant MM cell transcriptome signature, including AXL, may be a marker of disease progression and overall survival; and is even superior to many conventional biomarkers. It has been hypothesized that AXL could serve as a therapeutic target to reactivate the dormant cell population, making them more susceptible to chemotherapy and eradicate residual cancer cells in MM.

### 4.4. Clinical Trials Using AXL Inhibitors in Multiple Myeloma

Cabozantinib is an orally bioavailable multi-kinase inhibitor which targets vascular endothelial growth factor (VEGF) receptors, hepatocyte growth factor receptor (HGFR, also known as MET), AXL, and other receptor tyrosine kinases. For relapsed/refractory MM patients, two phase 1 studies were already conducted (NCT01866293, NCT01582295). Patients received treatment with an initial starting dose of 40 mg per day, however no significant single-agent activity could be observed [64]. A novel phase I/II trial of cabozantinib in combination with the proteasome inhibitor carfilzomib in refractory multiple myeloma patients (NCT03201250) is currently recruiting patients. To date, inhibitors specific to AXL have not been tested in MM patients.

## 5. The Role of AXL in Other Hematological Cancers

### 5.1. AXL in Chronic Lymphocytic Leukemia (CLL)

Chronic lymphocytic leukemia (CLL) is an incurable B-cell malignancy characterized by a progressive accumulation of monoclonal B lymphocytes in the bone marrow, lymph nodes and peripheral blood. AXL phosphorylation was found constitutively expressed on CLL-B cells and its expression is an independent prognostic factor in CLL patients [49]. Ghosh et al. found that expression of p-AXL was correlated with other phosphorylated kinases including Lyn, phosphoinositide-3 kinase, SyK/ζ-associated protein (70 kDa) and phospholipase C-γ2 in CLL B-cells [65]. AXL was found to regulate CLL B-cell survival by activation of fibroblast growth factor receptor (FGFR) signaling through complex formation with FGFR3 [66]. Targeting AXL with BGB324 or TP-0903, specific AXL inhibitors, induced apoptosis in a dose- and time-dependent manner [65,67]. AXL inhibition reduced expression of the anti-apoptotic protein myeloid cell leukemia 1 (Mcl-1), B-cell lymphoma 2 (Bcl-2), and X-linked inhibitor of apoptosis protein (XIAP), and increased expression of the pro-apoptotic protein BIM (Bcl-2-like protein 11) [67]. In addition, bone marrow stromal cells could not abrogate the apoptotic effect. Boysen et al. found that p53 activation negatively regulates AXL expression via upregulation of miR-34a in CLL B-cells and it has been suggested that p53-inactivation stabilizes the AXL protein in CLL [68]. Therapeutically, the combination of AXL inhibitors and Bruton’s tyrosine kinase (BTK) inhibitors, including ibrutinib, has been extensively studied. CLL B-cells obtained from ibrutinib-treated patients were found to be highly sensitive to AXL inhibition in vitro [69]. Preclinical combination of AXL inhibitors and ibrutinib significantly induced apoptosis compared to single agent therapy [70].

### 5.2. AXL in Chronic Myeloid Leukemia (CML)

Chronic myeloid leukemia (CML) is a hematopoietic stem cell disorder and is characterized by the presence of the Philadelphia (Ph1) chromosome. Imatinib, an anti-Abl (the Abelson proto-oncogene) tyrosine kinase inhibitor, is frequently used as a first-line treatment for CML patients and AXL is overexpressed in imatinib resistant CML cell lines and patients [71]. Gioia et al. demonstrated that AXL also regulates nilotinib-resistance, a second generation Abl inhibitor [72]. Depletion of Casitas B-lineage Lymphoma (CBL), an E3-ubiquitin ligase, resulted in increased Axl and Lyn expression and promoted nilotinib-resistance. On the other hand, increased CBL expression reduced Axl and Lyn expression and resensitized the cells to drug treatment. Surprisingly, authors found that the role of AXL in drug resistance was independent of its ligand GAS6. In addition, Sodare et al. demonstrated that imatinib-resistance can be partially reversed by decreasing Axl expression through *ZNF224* (zinc finger protein 224) upregulation [73]. Besides its role in drug resistance, AXL is highly expressed in primary CD34+ leukemia stem cells and its expression is dependent on the breakpoint cluster region/abelson murine leukemia viral oncogene homolog 1 (BCR-ABL) protein level rather than its tyrosine kinase activity [74]. Axl silencing significantly decreased the survival and self-renewal capacity of human CD34+ CML cells, while it has no effect on normal bone marrow CD34+ cells. In vivo, AXL inhibition resulted in prolonged survival of tumor-bearing mice and reduced the growth of CD34+ leukemia stem cells. Furthermore, they found that GAS6/AXL ligation stabilized beta-catenin levels, an important regulator of self-renewal, in CD34+ CML cells. In vivo treatment with BGB324 reduced tumor growth in BCR-ABL1 T315I-mutated (resistant to all approved agents) and ponatinib-resistant preclinical mouse models [75]. Additive effects with imatinib via inhibition of Stat5 activity has been demonstrated as well.

### 5.3. AXL in Acute Myeloid Leukemia (AML)

Acute myeloid leukemia (AML) is a clonal disease of hematopoietic progenitors characterized by immature myeloid cell proliferation and bone marrow failure. AXL was first found to be activated in AML cells in 1994 [75], and later studies revealed that AXL represents an independent prognostic factor and therapeutic target in AML patients [76]. AXL is highly expressed in the mononucleated cells, blasts, and leukemic stem cells of AML patients and is correlated with a poor prognosis, whereas other TAM receptors lacked prognostic relevance in AML patients [76,77]. GAS6 has been reported to be an adverse prognostic marker in de novo cytogenetically normal AML [78]. Ben-Batalla et al. highlighted the importance of GAS6/AXL signaling in proliferation, survival, and chemoresistance of AML cells [77]. AML cells induced GAS6 expression and secretion by bone marrow stromal cells, which in turn increased AXL activation. This paracrine interaction between both cell types creates a chemoprotective cancer cell niche. BGB324 treatment of AML cells inhibited Akt and Erk signaling, increased pro-apoptotic protein Puma and reduced anti-apoptotic Bcl-2 protein expression. In vivo, BGB324 treatment restored chemosensitivity of AML cells to doxorubicin (Doxo) [77].

About 25–30% of AML patients harbor internal tandem duplication (ITD) mutations in the Fms-like tyrosine kinase-3 (FLT3) [79]. The presence of FLT3-ITD correlates with a poor prognosis and AXL phosphorylation has been associated with FLT3 activation [44]. Soluble AXL chimeric protein (AXL-Fc) and the pharmacological AXL inhibitor Foretinib (XL-880) were able to inhibit cell growth, relieve the myeloid differentiation block, and induce cell cycle-arrest and apoptosis of FLT3-ITD+ AML cells. In addition, Park et al. [44] demonstrated that AXL expression is required for resistance to FLT3-targeted therapies in AML. It has been suggested that treatment with FLT3 inhibitor Midostaurin (PKC412) increases AXL phosphorylation and gives a survival advantage to a subset of resistant AML cells. Recently, Dumas et al. demonstrated that the hematopoietic niche induces AXL overexpression and protects FLT3-ITD AML cells against FLT3-targeted therapies including quizartinib [80]. They found that bone marrow derived cytokines sustained signal transducer and activator of transcription 5 (STAT5) phosphorylation in quizartinib-treated cells and together with the hypoxic microenvironment contributed to enhanced AXL expression [80]. Importantly, lentiviral knockdown of Axl restored sensitivity towards FLT3 inhibitor Quizartinib (AC220) in vivo. Moreover, BGB324 demonstrated anti-tumor activity in FLT3 wild-type as well as FLT3-mutated AML cells [77].

### 5.4. AXL in Mantle Cell Lymphoma (MCL)

Mantle cell lymphoma (MCL) is a rare B-cell cancer that carries a relatively poor prognosis compared to other forms of non-Hodgkin’s lymphoma. MCL is characterized by cyclin D1 overexpression and the cytogenetic abnormality t(11;14)(q13;q32) [81]. AXL inhibitor BGB324 decreased cell proliferation and induced cell-cycle arrest and apoptosis in MCL cell lines and patient cells in vitro. Besides, BGB324 also reduced cyclin D1, β-catenin, Akt, and NF-kB activity. Ibrutinib treatment in combination with the AXL inhibitor BGB324 showed synergetic effects on MCL apoptosis in vitro. Furthermore, in a xenograft mouse model of MCL, treatment with BGB324 suppressed tumor growth with a higher efficacy than ibrutinib.

More recently, the role of AXL inhibition on Chimere Antigen Receptor T-cell (CART) function was investigated. TP0903 treatment resulted in the polarization of CARTs into a Th1 phenotype when T cells were stimulated with Jeko, a CD19+ MCL cell line [82]. AXL inhibition significantly reduced the expression of inhibitory receptors programmed cell death-1 (PD-1) and lymphocyte-activation gene 3 (LAG3) on activated CARTs. In vitro, AXL inhibition significantly reduced the number of regulatory T cells, while no effect could be observed on the number of effector T cells. The transcriptome of activated CARTs treated with TP0903 was compared to untreated CARTs and more than 100 genes were differentially expressed. Genes related to cell junction, cell migration and immune synapse were significantly upregulated in TP0903-treated CARTs. To investigate the effect of AXL inhibition on CARTs in vivo, mice were inoculated with Jeko tumor cells, treated with CD19-directed CARTs in combination with or without TP0903 and rechallenged with Jeko cells. Mice that received both CARTs and AXL inhibitors rejected the tumor, while mice receiving only CARTs redeveloped MCL. These data suggest that AXL inhibition supports CART cell persistence in vivo.

### 5.5. AXL in T Cell Lymphoma

T cell lymphomas are a heterogeneous group of malignancies characterized by the expansion of a malignant T cell clone. In T cell lymphoma, AXL expression is very low [83]. However, Lee et al. demonstrated that overexpression of AXL induced LIGHT upregulation. LIGHT, also known as tumor necrosis factor superfamily member 14 (TNFSF14), is a TNF superfamily ligand that regulates apoptosis, increases T cell proliferation and cytokine production, and induces dendritic cell maturation. AXL-overexpressing T lymphoma cells increased the susceptibility to cytotoxic T cells (CTL)- and NK cell-mediated cell lysis. EL4-AXL-bearing mice had a reduced tumor volume compared to control mice due to increased apoptotic cell death. In addition, increased infiltration of cytotoxic T cells and natural killer cells was observed in EL4-AXL-bearing mice. The authors suggest a possible tumor suppressor role for AXL in T cell lymphoma by upregulation of LIGHT. However, we still need to consider that AXL is rather low or absent in T cell lymphomas and has been overexpressed in this specific cell type. This study highlights again the diverse role of AXL in different cell types, but whether it can function as a tumor suppressor is still unclear.

## 6. Targeting AXL and Its Therapeutic Potential in Hematological Cancers

### 6.1. Specific AXL Inhibitors

BGB324 (R428/bemcentinib), is a first-in-class oral AXL kinase inhibitor [84]. BGB324 activity is limited to the tyrosine kinase subfamily and among all the 133 kinases, AXL is the most potently inhibited [85]. BGB324 has minimal off-target anti-proliferative or cytotoxic activity in two-dimensional assays in several cell types including primary dendritic cells and T cells. Chen et al. found that BGB324 blocked lysosomal acidification and recycling, induced accumulation of autophagosomes and lysosomes, and increased tumor cell apoptosis [86]. Inhibition of autophagy by autophagy inhibitors or autophagic gene-knockout alleviated the BGB324-induced vacuole formation and apoptosis. Expression of granulocyte macrophage colony-stimulating factor (GM-CSF), IL-1β, IL-6, and macrophage inflammatory protein-1α were found to be reduced in a dose-dependent manner in primary tumor lysates from BGB324-treated animals [85]. In this study, the safety of BGB324 was demonstrated after long-term dosing as well as in a 14 day tolerability study. BGB324 has been tested in a phase II clinical trial (NCT02488408) and included patients with relapsed/refractory AML or myeloid dysplastic syndrome (MDS). Loges et al. reported that BGB324 can be safely administered for prolonged periods at doses that abrogate AXL signaling and exhibit anti-leukemic activity [87].

TP-0903 is another high-affinity orally bioavailable AXL inhibitor which also inhibits FLT3 activity. This inhibitor increased apoptosis of primary CLL B-cells, even if the patient had high risk factors (e.g., 17p/P53 deletions) or progressed on other agents including ibrutinib [67,70]. Moreover, TP-0903 showed promising in vitro and in vivo activity in de novo and drug resistant FLT3-ITD+ AML [88]. A phase 1/2 clinical trial of TP-0903 in patients with previously treated CLL (NCT03572634) is currently recruiting patients.

Kariolis et al. engineered an AXL “decoy receptor” that binds GAS6 with high affinity and inhibits its function [89]. This AXL variant contains four mutations and has an 80-fold improved affinity compared to the wild-type AXL receptor. Using this decoy receptor, GAS6 could be effectively sequestrated whereby AXL signaling is specifically disturbed. It has been demonstrated that the improved decoy receptor MYD1-72 significantly reduced cell growth and induced cytotoxicity in both OCI-AML3 and MV4:11 cells [90].

### 6.2. Multi-Target AXL Inhibitors

There is a broad range of orally bioavailable AXL inhibitors that also inhibit other kinases including the TAM family members TYRO3 and MERTK; as well as other RTKs such as MET, FLT3, Recepteur d’Origine Nantais (RON), and AURORA A/B. Gilteritinib inhibits FLT3, AXL, and anaplastic lymphoma kinase (ALK or CD246) and is FDA approved for the treatment of relapsed/refractory FLT3-mutated AML patients [91]. Merestinib (LY2801653) is a multikinase inhibitor and targets MET, macrophage-stimulating protein receptor (MST1R, also known as RON), FLT3, AXL, MERTK, angiopoietin-1 receptor (also known as CD202B or TEK), proto-oncogene tyrosine-protein kinase ROS (ROS1), discoidin domain receptor family member 1/2 (DDR1/2), and the MAP kinase-interacting serine/threonine-protein kinases 1/2 (MKNK1/2) [92]. Merestinib potently exhibited anti-leukemic effects in a xenograft mouse model of AML [93]. Currently, a phase 1 clinical trial in relapsed/refractory AML patients is ongoing (NCT03125239). Cabozantinib (XL184) targets VEGFR2, c-MET, KIT (also known as CD117), AXL, and FLT3; and induces apoptosis in FLT3-ITD+ leukemia cells in a dose-dependent manner [94,95]. However, FLT3-ITD lacking leukemia cell lines were resistant to cabozantinib [95]. In a clinical trial (NCT01961765), cabozantinib was well tolerated in AML patients and was a potent inhibitor of FLT3/ITD altered tyrosine kinases [96]. A phase 1 clinical trial for multiple tumors including refractory AML is still ongoing (NCT03878524). In Table 1, the currently available AXL inhibitors, their targets and clinical trials are summarized.

## 7. Conclusions and Future Developments

Over the past 5 years, the role of AXL in normal cell biology and cancer cells has been widely investigated. AXL is expressed by various cell types and regulates numerous processes including survival, proliferation, angiogenesis, and immune modulation. The role of AXL in the immune system is clearly paradoxical and is cell type dependent. Its activation can either induce or inhibit the expression of pro-inflammatory cytokines, resulting in a pro- or anti-tumor immune response. This is one potential concern that needs to be further evaluated in preclinical models and clinical trials.

In hematological malignancies, AXL is generally correlated with a poor prognosis; however, its role in chemoresistance and dormancy is cancer cell specific and remains poorly understood. AXL inhibitors decrease survival and induce apoptosis in AXL expressing CML, CLL, and AML cells; while combination therapies with standard-of-care agents are even more successful. In MM, AXL is a potential mediator of myeloma cell dormancy and it has been hypothesized that AXL targeting would reactive dormant myeloma cells, sensitizing them to chemotherapeutic agents. Combination studies with AXL inhibitors and chemotherapeutic agents using the in vivo dormancy model are recommended to further investigate its therapeutic potential. As the bone marrow microenvironment plays a crucial role in MM pathogenesis, it is recommended to further evaluate the role of AXL in distinct cell types of the bone marrow including bone marrow stromal cells, endothelial cells, M2 macrophages, and dendritic cell subtypes.

In the last year, there has been growing interest to further unravel the role of AXL in cancer cell biology and to combine it specifically with immune checkpoint inhibitors and/or chemotherapeutic agents to induce a proper anti-tumor response. The ongoing clinical trials, development of even more specific inhibitors, and new combination strategies, will determine the future direction of AXL inhibitors in the treatment of hematological cancers.

## Figures and Tables

**Table 1:** AXL inhibitors in preclinical and clinical development of hematological cancers.

Drug	Targets	IC50 AXL/MERTK	Disease	Phase
**TP-0903**	Axl [70,97] (Aurr A and B, Jak2, Alk, Abl)	27 nM/−	Previously Treated CLL	I/II
**BGB324**	Axl [85,98] (Abl, Mertk, Tyro3, Her-2, EGFR, InsR, PDGFR-β)	14 nM/−	AML or MDS	Ib/II
**MYD1-72**	Axl [90]	0.7 nM/−	AML	Preclinical
**Merestinib**	Met/Tek/ROS/Axl/DDR1/2/Flt3 [92,93]	11 nM/2 nM	Relapsed/Refractory AML	I
**Cabozantinib**	VEGFR2, Flt3, Met, KIT, Ret, and Axl [64]	7 nM/1.3 nM	Relapsed/Refractory AML Refractory MM	I/II
**Gilteritinib**	Flt3/Axl [93]	0.73 nM/−	AML (FLT3 mutated/relapsed/refractory/newly diagnosed)	I/II/III
**BMS777607**	Met, Ron, Axl, Tyro3 [93]	1.1 nM/3.9 nM	MM	Preclinical
**XL880**	Met, Axl, Vegfr2, Pdgfrβ and Tie2 [44]	11 nM/0.4 nM	AML	Preclinical

Abbreviations: Aurr = Aurora kinases, Jak2 = Janus Kinase 2, Alk = Anaplastic lymphoma kinase, Abl = Abelson murine leukemia viral oncogene homolog 1, Mertk = Proto-oncogene tyrosine-protein kinase MER, Tyro3 = tyrosine-protein kinase receptor 3, InsR = Insulin Receptor, PDGFR = platelet-derived growth factor receptor, Met = hepatocyte growth factor receptor, Tek = angiopoietin-1 receptor, ROS = proto-oncogene tyrosine-protein kinase ROS, DDR = discoidin domain receptor family member 1/2, VEGFR = vascular endothelial growth factor receptor, FLT = fms like tyrosine kinase, KIT = CD117, Ret = rearranged during transfection, Ron = Recepteur d’Origine Nantais, Tie2 = tyrosine kinase with immunoglobulin-like and EGF-like domains 2.

## References

[1] O’Bryan J.P., Frye R.A., Cogswell P.C., Neubauer A., Kitch B., Prokop C., Espinosa R., Le Beau M.M., Earp H.S., Liu E.T. (1991). Axl, a transforming gene isolated from primary human myeloid leukemia cells, encodes a novel receptor tyrosine kinase. Mol. Cell. Biol..

[2] Lai C., Gore M., Lemke G. (1994). Structure, Expression, and Activity of Tyro-3, a Neural Adhesion-Related Receptor Tyrosine Kinase. Oncogene.

[3] Heiring C., Dahlback B., Muller Y.A. (2004). Ligand recognition and homophilic interactions in Tyro3: Structural insights into the Axl/Tyro3 receptor tyrosine kinase family. J. Biol. Chem..

[4] Sasaki T., Knyazev P.G., Clout N.J., Cheburkin Y., Gohring W., Ullrich A., Timpl R., Hohenester E. (2006). Structural basis for Gas6-Axl signalling. EMBO J..

[5] Sasaki T., Knyazev P.G., Cheburkin Y., Gohring W., Tisi D., Ullrich A., Timpl R., Hohenester E. (2002). Crystal structure of a C-terminal fragment of growth arrest-specific protein Gas6. Receptor tyrosine kinase activation by laminin G-like domains. J. Biol. Chem..

[6] Stitt T.N., Conn G., Gore M., Lai C., Bruno J., Radziejewski C., Mattsson K., Fisher J., Gies D.R., Jones P.F. (1995). The anticoagulation factor protein S and its relative, Gas6, are ligands for the Tyro 3/Axl family of receptor tyrosine kinases. Cell.

[7] Manfioletti G., Brancolini C., Avanzi G., Schneider C. (1993). The Protein Encoded by a Growth Arrest-Specific Gene (Gas6) Is a New Member of the Vitamin-K-Dependent Proteins Related to Protein-S, a Negative Coregulator in the Blood-Coagulation Cascade. Mol. Cell. Biol..

[8] Avanzi G.C., Gallicchio M., Cavalloni G., Gammaitoni L., Leone F., Rosina A., Boldorini R., Monga G., Pegoraro L., Varnum B. (1997). GAS6, the ligand of Axl and Rse receptors, is expressed in hematopoietic tissue but lacks mitogenic activity. Exp. Hematol..

[9] Nakano T., Higashino K., Kikuchi N., Kishino J., Nomura K., Fujita H., Ohara O., Arita H. (1995). Vascular smooth muscle cell-derived, Gla-containing growth-potentiating factor for Ca(2+)-mobilizing growth factors. J. Biol. Chem..

[10] Braunger J., Schleithoff L., Schulz A.S., Kessler H., Lammers R., Ullrich A., Bartram C.R., Janssen J.W. (1997). Intracellular signaling of the Ufo/Axl receptor tyrosine kinase is mediated mainly by a multi-substrate docking-site. Oncogene.

[11] Cosemans J.M., Van Kruchten R., Olieslagers S., Schurgers L.J., Verheyen F.K., Munnix I.C., Waltenberger J., Angelillo-Scherrer A., Hoylaerts M.F., Carmeliet P. (2010). Potentiating role of Gas6 and Tyro3, Axl and Mer (TAM) receptors in human and murine platelet activation and thrombus stabilization. J. Thromb. Haemost..

[12] Couchie D., Lafdil F., Martin-Garcia N., Laperche Y., Zafrani E.S., Mavier P. (2005). Expression and role of Gas6 protein and of its receptor Axl in hepatic regeneration from oval cells in the rat. Gastroenterology.

[13] Park I.K., Giovenzana C., Hughes T.L., Yu J.H., Trotta R., Caligiuri M.A. (2009). The Axl/Gas6 pathway is required for optimal cytokine signaling during human natural killer cell development. Blood.

[14] Pierce A., Bliesner B., Xu M., Nielsen-Preiss S., Lemke G., Tobet S., Wierman M.E. (2008). Axl and Tyro3 modulate female reproduction by influencing gonadotropin-releasing hormone neuron survival and migration. Mol. Endocrinol..

[15] Tang H., Chen S., Wang H., Wu H., Lu Q., Han D. (2009). TAM receptors and the regulation of erythropoiesis in mice. Haematologica.

[16] Rothlin C.V., Ghosh S., Zuniga E.I., Oldstone M.B.A., Lemke G. (2007). TAM receptors are pleiotropic inhibitors of the innate immune response. Cell.

[17] Caraux A., Lu Q.X., Fernandez N., Riou S., Di Santo J.P., Raulet D.H., Lemke G., Roth C. (2006). Natural killer cell differentiation driven by Tyro3 receptor tyrosine kinases. Nat. Immunol..

[18] Kim E.M., Lee E.H., Lee H.Y., Choi H.R., Ji K.Y., Kim S.M., Kim K.D., Kang H.S. (2017). Axl signaling induces development of natural killer cells in vitro and in vivo. Protoplasma.

[19] Budagian V., Bulanova E., Orinska Z., Thon L., Mamat U., Bellosta P., Basilico C., Adam D., Paus R., Bulfone-Paus S. (2005). A promiscuous liaison between IL-15 receptor and Axl receptor tyrosine kinase in cell death control (Retracted Article. See vol 30, pg 627, 2011). EMBO J..

[20] Scutera S., Fraone T., Musso T., Cappello P., Rossi S., Pierobon D., Orinska Z., Paus R., Bulfone-Paus S., Giovarelli M. (2009). Survival and Migration of Human Dendritic Cells Are Regulated by an IFN-alpha-Inducible Axl/Gas6 Pathway. J. Immunol..

[21] Han J., Bae J., Choi C.Y., Choi S.P., Kang H.S., Jo E.K., Park J., Lee Y.S., Moon H.S., Park C.G. (2016). Autophagy induced by AXL receptor tyrosine kinase alleviates acute liver injury via inhibition of NLRP3 inflammasome activation in mice. Autophagy.

[22] Fujimori T., Grabiec A.M., Kaur M., Bell T.J., Fujino N., Cook P.C., Svedberg F.R., MacDonald A.S., Maciewicz R.A., Singh D. (2015). The Axl receptor tyrosine kinase is a discriminator of macrophage function in the inflamed lung. Mucosal Immunol..

[23] Grabiec A.M., Goenka A., Fife M.E., Fujimori T., Hussell T. (2018). Axl and MerTK receptor tyrosine kinases maintain human macrophage efferocytic capacity in the presence of viral triggers. Eur. J. Immunol..

[24] Zhao G.J., Zheng J.Y., Bian J.L., Chen L.W., Dong N., Yu Y., Hong G.L., Chandoo A., Yao Y.M., Lu Z.Q. (2017). Growth Arrest-Specific 6 Enhances the Suppressive Function of CD4(+) CD25(+) Regulatory T Cells Mainly through Axl Receptor. Mediat. Inflamm..

[25] Paccez J.D., Vasques G.J., Correa R.G., Vasconcellos J.F., Duncan K., Gu X., Bhasin M., Libermann T.A., Zerbini L.F. (2013). The receptor tyrosine kinase Ax1 is an essential regulator of prostate cancer proliferation and tumor growth and represents a new therapeutic target. Oncogene.

[26] Shiozawa Y., Pedersen E.A., Patel L.R., Ziegler A.M., Havens A.M., Jung Y.H., Wang J.C., Zalucha S., Loberg R.D., Pienta K.J. (2010). GAS6/AXL Axis Regulates Prostate Cancer Invasion, Proliferation, and Survival in the Bone Marrow Niche. Neoplasia.

[27] Martinelli E., Martini G., Cardone C., Troiani T., Liguori G., Vitagliano D., Napolitano S., Morgillo F., Rinaldi B., Melillo R.M. (2015). AXL is an oncotarget in human colorectal cancer. Oncotarget.

[28] Sawabu T., Seno H., Kawashima T., Fukuda A., Uenoyama Y., Kawada M., Kanda N., Sekikawa A., Fukui H., Yanagita M. (2007). Growth arrest-specific gene 6 and Axl signaling enhances gastric cancer cell survival via Akt pathway. Mol. Carcinogen..

[29] Axelrod H., Pienta K.J. (2014). Axl as a mediator of cellular growth and survival. Oncotarget.

[30] Woo S.M., Min K.J., Seo S.U., Kim S., Kubatka P., Park J.W., Kwon T.K. (2019). Axl Inhibitor R428 Enhances TRAIL-Mediated Apoptosis Through Downregulation of c-FLIP and Survivin Expression in Renal Carcinoma. Int. J. Mol. Sci..

[31] Goruppi S., Chiaruttini C., Ruaro M.E., Varnum B., Schneider C. (2001). Gas6 induces growth, beta-catenin stabilization, and T-cell factor transcriptional activation in contact-inhibited C57 mammary cells. Mol. Cell. Biol..

[32] Goruppi S., Ruaro E., Schneider C. (1996). Gas6, the ligand of Axl tyrosine kinase receptor, has mitogenic and survival activities for serum starved NIH3T3 fibroblasts. Oncogene.

[33] Goruppi S., Ruaro E., Varnum B., Schneider C. (1997). Requirement of phosphatidylinositol 3-kinase-dependent pathway and Src for Gas6-Axl mitogenic and survival activities in NIH 3T3 fibroblasts. Mol. Cell. Biol..

[34] Han J., Tian R., Yong B.C., Luo C.Q., Tan P.X., Shen J.N., Peng T.S. (2013). Gas6/Axl mediates tumor cell apoptosis, migration and invasion and predicts the clinical outcome of osteosarcoma patients. Biochem. Bioph. Res. Commun..

[35] Vajkoczy P., Knyazev P., Kunkel A., Capelle H.H., Behrndt S., von Tengg-Kobligk H., Kiessling F., Eichelsbacher U., Essig M., Read T.A. (2006). Dominant-negative inhibition of the Axl receptor tyrosine kinase suppresses brain tumor cell growth and invasion and prolongs survival. Proc. Natl. Acad. Sci. USA.

[36] Nakano T., Tani M., Ishibashi Y., Kimura K., Park Y.B., Imaizumi N., Tsuda H., Aoyagi K., Sasaki H., Ohwada S. (2003). Biological properties and gene expression associated with metastatic potential of human osteosarcoma. Clin. Exp. Metastas..

[37] Revach O.Y., Sandler O., Samuels Y., Geiger B. (2019). Cross-Talk between Receptor Tyrosine Kinases AXL and ERBB3 Regulates Invadopodia Formation in Melanoma Cells. Cancer Res..

[38] Li W.J., Xiong X.H., Abdalla A., Alejo S., Zhu L.Y., Lu F., Sun H. (2018). HGF-induced formation of the MET-AXL-ELMO2-DOCK180 complex promotes RAC1 activation, receptor clustering, and cancer cell migration and invasion. J. Biol. Chem..

[39] Duan Y.T., Luo L.L., Qiao C.X., Li X.Y., Wang J., Liu H., Zhou T.T., Shen B.F., Lv M., Feng J.N. (2019). A novel human anti-AXL monoclonal antibody attenuates tumour cell migration. Scand. J. Immunol..

[40] Hong C.C., Lay J.D., Huang J.S., Cheng A.L., Tang J.L., Lin M.T., Lai G.M., Chuang S.E. (2008). Receptor tyrosine kinase AXL is induced by chemotherapy drugs and overexpression of AXL confers drug resistance in acute myeloid leukemia. Cancer Lett..

[41] Wang C., Jin H., Wang N., Fan S., Wang Y., Zhang Y., Wei L., Tao X., Gu D., Zhao F. (2016). Gas6/Axl Axis Contributes to Chemoresistance and Metastasis in Breast Cancer through Akt/GSK-3beta/beta-catenin Signaling. Theranostics.

[42] Quinn J.M., Greenwade M.M., Palisoul M.L., Opara G., Massad K., Zhao P.N., Beck-Noia H., Hagemann I.S., Hagemann A.R., McCourt C.K. (2019). Therapeutic Inhibition of the Receptor Tyrosine Kinase AXL Improves Sensitivity to Platinum and Taxane in Ovarian Cancer. Mol. Cancer Ther..

[43] D’Errico G., Alonso-Nocelo M., Vallespinos M., Hermann P.C., Alcala S., Garcia C.P., Martin-Hijano L., Valle S., Earl J., Cassiano C. (2019). Tumor-associated macrophage-secreted 14-3-3 zeta signals via AXL to promote pancreatic cancer chemoresistance. Oncogene.

[44] Park I.K., Mishra A., Chandler J., Whitman S.P., Marcucci G., Caligiuri M.A. (2013). Inhibition of the receptor tyrosine kinase Axl impedes activation of the FLT3 internal tandem duplication in human acute myeloid leukemia: Implications for Axl as a potential therapeutic target. Blood.

[45] Badarni M., Prasad M., Balaban N., Zorea J., Yegodayev K.M., Joshua B.Z., Dinur A.B., Grenman R., Rotblat B., Cohen L. (2019). Repression of AXL expression by AP-1/JNK blockage overcomes resistance to PI3Ka therapy. JCI Insight.

[46] Elkabets M., Pazarentzos E., Juric D., Sheng Q., Pelossof R.A., Brook S., Benzaken A.O., Rodon J., Morse N., Yan J.J. (2015). AXL mediates resistance to PI3Kalpha inhibition by activating the EGFR/PKC/mTOR axis in head and neck and esophageal squamous cell carcinomas. Cancer Cell.

[47] Brand T.M., Iida M., Stein A.P., Corrigan K.L., Braverman C.M., Luthar N., Toulany M., Gill P.S., Salgia R., Kimple R.J. (2014). AXL mediates resistance to cetuximab therapy. Cancer Res..

[48] Taniguchi H., Yamada T., Wang R., Tanimura K., Adachi Y., Nishiyama A., Tanimoto A., Takeuchi S., Araujo L.H., Boroni M. (2019). AXL confers intrinsic resistance to osimertinib and advances the emergence of tolerant cells. Nat. Commun..

[49] Zhang Z.F., Lee J.C., Lin L.P., Olivas V., Au V., LaFramboise T., Abdel-Rahman M., Wang X.Q., Levine A.D., Rho J.K. (2012). Activation of the AXL kinase causes resistance to EGFR-targeted therapy in lung cancer. Nat. Genet..

[50] Yumoto K., Eber M.R., Wang J.C., Cackowski F.C., Decker A.M., Lee E., Nobre A.R., Aguirre-Ghiso J.A., Jung Y.H., Taichman R.S. (2016). Axl is required for TGF-beta 2-induced dormancy of prostate cancer cells in the bone marrow. Sci. Rep..

[51] Axelrod H.D., Valkenburg K.C., Amend S.R., Hicks J.L., Parsana P., Torga G., DeMarzo A.M., Pienta K.J. (2019). AXL Is a Putative Tumor Suppressor and Dormancy Regulator in Prostate Cancer. Mol. Cancer Res..

[52] Chiu K.C., Lee C.H., Liu S.Y., Chou Y.T., Huang R.Y., Huang S.M., Shieh Y.S. (2015). Polarization of tumor-associated macrophages and Gas6/Axl signaling in oral squamous cell carcinoma. Oral Oncol..

[53] Lai S., Li R., Raha P., Hu Y., Yan J., Zhang H., Marotta A., Zhang Z. (2018). Abstract B148: Activity of the TAM kinase-targeting compound, SLC-391, is mediated by the engagement of the immune system in CT-26 syngeneic mouse model. Mol. Cancer Ther..

[54] Aguilera T.A., Rafat M., Castellini L., Shehade H., Kariolis M.S., Hui A.B.Y., Stehr H., von Eyben R., Jiang D., Ellies L.G. (2016). Reprogramming the immunological microenvironment through radiation and targeting Axl. Nat. Commun..

[55] Tsukita Y., Fujino N., Miyauchi E., Saito R., Fujishima F., Itakura K., Kyogoku Y., Okutomo K., Yamada M., Okazaki T. (2019). Axl kinase drives immune checkpoint and chemokine signalling pathways in lung adenocarcinomas. Mol. Cancer.

[56] Guo Z.Q., Li Y., Zhang D.D., Ma J.Y. (2017). Axl inhibition induces the antitumor immune response which can be further potentiated by PD-1 blockade in the mouse cancer models. Oncotarget.

[57] Yokoyama Y., Lew E.D., Seelige R., Tindall E.A., Walsh C., Fagan P.C., Lee J.Y., Nevarez R., Oh J., Tucker K.D. (2019). Immuno-oncological Efficacy of RXDX-106, a Novel TAM (TYRO3, AXL, MER) Family Small-Molecule Kinase Inhibitor. Cancer Res..

[58] Kumar S.K., Rajkumar V., Kyle R.A., van Duin M., Sonneveld P., Mateos M.V., Gay F., Anderson K.C. (2017). Multiple myeloma. Nat. Rev. Dis. Primers.

[59] Dhodapkar M.V. (2016). MGUS to myeloma: A mysterious gammopathy of underexplored significance. Blood.

[60] Chim C.S., Kumar S.K., Orlowski R.Z., Cook G., Richardson P.G., Gertz M.A., Giralt S., Mateos M.V., Leleu X., Anderson K.C. (2018). Management of relapsed and refractory multiple myeloma: Novel agents, antibodies, immunotherapies and beyond. Leukemia.

[61] Waizenegger J.S., Ben-Batalla I., Weinhold N., Meissner T., Wroblewski M., Janning M., Riecken K., Binder M., Atanackovic D., Taipaleenmaeki H. (2015). Role of Growth arrest-specific gene 6-Mer axis in multiple myeloma. Leukemia.

[62] Engelmann J., Ben Batalla I., Taipaleenmaki H., Riecken K., Gensch V., Paesler S., Berenbrok N., Vargas M.E., Waizenegger J., Fehse B. (2018). Blockade of Mer By the Small Molecule Inhibitor R992 Inhibits Multiple Myeloma and Its Associated Bone Disease By Restoring the Perturbed Bone Homeostasis. Blood.

[63] Khoo W.H., Ledergor G., Weiner A., Roden D.L., Terry R.L., McDonald M.M., Chai R.C., De Veirman K., Owen K.L., Opperman K.S. (2019). A niche-dependent myeloid transcriptome signature defines dormant myeloma cells. Blood.

[64] Lendvai N., Yee A.J., Tsakos I., Alexander A., Devlin S.M., Hassoun H., Korde N., Lesokhin A.M., Landau H., Mailankody S. (2016). Phase IB study of cabozantinib in patients with relapsed and/or refractory multiple myeloma. Blood.

[65] Ghosh A.K., Secreto C., Boysen J., Sassoon T., Shanafelt T.D., Mukhopadhyay D., Kay N.E. (2011). The novel receptor tyrosine kinase Axl is constitutively active in B-cell chronic lymphocytic leukemia and acts as a docking site of nonreceptor kinases: Implications for therapy. Blood.

[66] Sinha S., Boysen J., Nelson M., Warner S.L., Bearss D., Kay N.E., Ghosh A.K. (2016). Axl activates fibroblast growth factor receptor pathway to potentiate survival signals in B-cell chronic lymphocytic leukemia cells. Leukemia.

[67] Sinha S., Boysen J., Nelson M., Secreto C., Warner S.L., Bearss D.J., Lesnick C., Shanafelt T.D., Kay N.E., Ghosh A.K. (2015). Targeted Axl Inhibition Primes Chronic Lymphocytic Leukemia B Cells to Apoptosis and Shows Synergistic/Additive Effects in Combination with BTK Inhibitors. Clin. Cancer Res..

[68] Boysen J., Sinha S., Price-Troska T., Warner S.L., Bearss D.J., Viswanatha D., Shanafelt T.D., Kay N.E., Ghosh A.K. (2014). The tumor suppressor axis p53/miR-34a regulates Axl expression in B-cell chronic lymphocytic leukemia: Implications for therapy in p53-defective CLL patients. Leukemia.

[69] Sinha S., Boysen J.C., Chaffee K.G., Kabat B.F., Slager S.L., Parikh S.A., Secreto C.R., Call T., Shanafelt T.D., Leis J.F. (2018). Chronic lymphocytic leukemia cells from ibrutinib treated patients are sensitive to Axl receptor tyrosine kinase inhibitor therapy. Oncotarget.

[70] Patel V., Keating M.J., Wierda W.G., Gandhi V. (2016). Preclinical combination of TP-0903, an AXL inhibitor and B-PAC-1, a procaspase-activating compound with ibrutinib in chronic lymphocytic leukemia. Leuk. Lymphoma.

[71] Ben-Batalla I., Erdmann R., Jorgensen H., Mitchell R., Ernst T., Von Amsberg G., Schafhausen P., Velthaus J.L., Rankin S., Clark R.E. (2017). Axl Blockade by BGB324 Inhibits BCR-ABL Tyrosine Kinase Inhibitor-Sensitive and -Resistant Chronic Myeloid Leukemia. Clin. Cancer Res..

[72] Gioia R., Tregoat C., Dumas P.Y., Lagarde V., Prouzet-Mauleon V., Desplat V., Sirvent A., Praloran V., Lippert E., Villacreces A. (2015). CBL controls a tyrosine kinase network involving AXL, SYK and LYN in nilotinib-resistant chronic myeloid leukaemia. J. Pathol..

[73] Sodaro G., Blasio G., Fiorentino F., Auberger P., Costanzo P., Cesaro E. (2018). ZNF224 is a transcriptional repressor of AXL in chronic myeloid leukemia cells. Biochimie.

[74] Jin Y.L., Nie D.N., Li J., Du X., Lu Y.H., Li Y.Q., Liu C., Zhou J.F., Pan J.X. (2017). Gas6/AXL Signaling Regulates Self-Renewal of Chronic Myelogenous Leukemia Stem Cells by Stabilizing beta-Catenin. Clin. Cancer Res..

[75] Neubauer A., Fiebeler A., Graham D.K., Obryan J.P., Schmidt C.A., Barckow P., Serke S., Siegert W., Snodgrass H.R., Huhn D. (1994). Expression of Axl, a Transforming Receptor Tyrosine Kinase, in Normal and Malignant Hematopoiesis. Blood.

[76] Rochlitz C., Lohri A., Bacchi M., Schmidt M., Nagel S., Fopp M., Fey M.F., Herrmann R., Neubauer A. (1999). Axl expression is associated with adverse prognosis and with expression of Bcl-2 and CD34 in de novo acute myeloid leukemia (AML): Results from a multicenter trial of the Swiss Group for Clinical Cancer Research (SAKK). Leukemia.

[77] Ben-Batalla I., Schultze A., Wroblewski M., Erdmann R., Heuser M., Waizenegger J.S., Riecken K., Binder M., Schewe D., Sawall S. (2013). Axl, a prognostic and therapeutic target in acute myeloid leukemia mediates paracrine crosstalk of leukemia cells with bone marrow stroma. Blood.

[78] Whitman S.P., Kohlschmidt J., Maharry K., Volinia S., Mrozek K., Nicolet D., Schwind S., Becker H., Metzeler K.H., Mendler J.H. (2014). GAS6 expression identifies high-risk adult AML patients: Potential implications for therapy. Leukemia.

[79] Gilliland D.G., Griffin J.D. (2002). The roles of FLT3 in hematopoiesis and leukemia. Blood.

[80] Dumas P.Y., Naudin C., Martin-Lanneree S., Izac B., Casetti L., Mansier O., Rousseau B., Artus A., Dufossee M., Giese A. (2019). Hematopoietic niche drives FLT3-ITD acute myeloid leukemia resistance to quizartinib via STAT5-and hypoxia-dependent upregulation of AXL. Haematologica.

[81] Gelebart P., Han J., Karlsen I., Gjerstad M.E., Helgeland L., Baran-Marszak F., Papp B., Cormack E.M. The AXL tyrosine kinase inhibitor, BGB324, induces cell death in mantle cell lymphoma. Proceedings of the EHA23 Library.

[82] Sakemura R., Yang N., Cox M.J., Sinha S., Hefazi M., Hansen M.J., Schick K.J., Forsman C.L., Boysen J.C., Tschumper R.C. (2018). Axl-RTK Inhibition Modulates T Cell Functions and Synergizes with Chimeric Antigen Receptor T Cell Therapy in B Cell Malignancies. Blood.

[83] Lee E.H., Kim E.M., Ji K.Y., Park A.R., Choi H.R., Lee H.Y., Kim S.M., Chung B.Y., Park C.H., Choi H.J. (2017). Axl acts as a tumor suppressor by regulating LIGHT expression in T lymphoma. Oncotarget.

[84] Sheridan C. (2013). First Axl inhibitor enters clinical trials. Nat. Biotechnol..

[85] Holland S.J., Pan A., Franci C., Hu Y.M., Chang B., Li W.Q., Duan M., Torneros A., Yu J.X., Heckrodt T.J. (2010). R428, a Selective Small Molecule Inhibitor of Axl Kinase, Blocks Tumor Spread and Prolongs Survival in Models of Metastatic Breast Cancer. Cancer Res..

[86] Chen F.F., Song Q.L., Yu Q. (2018). Axl inhibitor R428 induces apoptosis of cancer cells by blocking lysosomal acidification and recycling independent of Axl inhibition. Am. J. Cancer Res..

[87] Loges S., Gjertsen B.T., Heuser M., Ben-Batalla I., Micklem D., Jorg C., Kebenko M., Fiedler W.M., Cortes J.E. (2016). A first-in-patient phase I study of BGB324, a selective Axl kinase inhibitor in patients with refractory/relapsed AML and high-risk MDS. J. Clin. Oncol..

[88] Jeon J.Y., Park I.K., Buelow D.R., Whatcott C., Warner S.L., Blum W., Baker S. (2017). TP-0903, a Novel Axl Inhibitor with Activity in Drug Resistant FLT3-ITD+ AML through a Mechanism That Includes FLT3 Inhibition. Blood.

[89] Kariolis M.S., Miao Y.R., Ii D.S.J., Kapur S., Mathews I.I., Giaccia A.J., Cochran J.R. (2014). An engineered Axl ‘decoy receptor’ effectively silences the Gas6-Axl signaling axis. Nat. Chem. Biol..

[90] Kariolis M.S., Miao Y.R., Diep A., Nash S.E., Olcina M.M., Jiang D., Jones D.S., Kapur S., Mathews I.I., Koong A.C. (2017). Inhibition of the GAS6/AXL pathway augments the efficacy of chemotherapies. J. Clin. Investig..

[91] Dhillon S. (2019). Gilteritinib: First Global Approval. Drugs.

[92] Yan S.B., Peek V.L., Ajamie R., Buchanan S.G., Graff J.R., Heidler S.A., Hui Y.H., Huss K.L., Konicek B.W., Manro J.R. (2013). LY2801653 is an orally bioavailable multi-kinase inhibitor with potent activity against MET, MST1R, and other oncoproteins, and displays anti-tumor activities in mouse xenograft models. Invest. New Drugs.

[93] Kosciuczuk E.M., Saleiro D., Kroczynska B., Beauchamp E.M., Eckerdt F., Blyth G.T., Abedin S.M., Giles F.J., Altman J.K., Platanias L.C. (2016). Merestinib blocks Mnk kinase activity in acute myeloid leukemia progenitors and exhibits antileukemic effects in vitro and in vivo. Blood.

[94] Elisei R., Schlumberger M.J., Muller S.P., Schoffski P., Brose M.S., Shah M.H., Licitra L., Jarzab B., Medvedev V., Kreissl M.C. (2013). Cabozantinib in Progressive Medullary Thyroid Cancer. J. Clin. Oncol..

[95] Lu J.W., Wang A.N., Liao H.A., Chen C.Y., Hou H.A., Hu C.Y., Tien H.F., Ou D.L., Lin L.I. (2016). Cabozantinib is selectively cytotoxic in acute myeloid leukemia cells with FLT3-internal tandem duplication (FLT3-ITD). Cancer Lett..

[96] Fathi A.T., Blonquist T.M., Hernandez D. (2018). Cabozantinib is well tolerated in acute myeloid leukemia and effectively inhibits the resistance-conferring FLT3/tyrosine kinase domain/F691 mutation (vol 124, pg 306, 2018). Cancer Am. Cancer Soc..

[97] Shen Y.Y., Chen X.G., He J., Liao D.F., Zu X.Y. (2018). Axl inhibitors as novel cancer therapeutic agents. Life Sci..

[98] Vouri M., An Q., Birt M., Pilkington G.J., Hafizi S. (2015). Small molecule inhibition of Axl receptor tyrosine kinase potently suppresses multiple malignant properties of glioma cells. Oncotarget.

